# Enterohaemorrhagic *Escherichia coli* haemolysin is cleaved and inactivated by serine protease EspPα

**DOI:** 10.1111/j.1462-2920.2011.02431.x

**Published:** 2011-05

**Authors:** Jens Brockmeyer, Thomas Aldick, Jens Soltwisch, Wenlan Zhang, Philip I Tarr, André Weiss, Klaus Dreisewerd, Johannes Müthing, Martina Bielaszewska, Helge Karch

**Affiliations:** 1 Institute of Food Chemistry, University of MünsterCorrensstr. 45, 48149 Münster, Germany; 2 Institute of Hygiene, University of MünsterRobert-Koch-Str. 41, 48149 Münster, Germany; 3 Institute of Medical Physics and Biophysics, University of MünsterRobert-Koch-Str. 31, 48149 Münster, Germany; 4 Department of Pediatrics, Washington University School of MedicineCampus Box 8208, 660 S. Euclid, St. Louis, MO 63105, USA

## Abstract

The haemolysin from enterohaemorrhagic *Escherichia coli* (EHEC-Hly) and the serine protease EspPα are putative virulence factors of EHEC. We investigated the interplay between these secreted factors and demonstrate that EspPα cleaves the 107 kDa large EHEC-Hly. Degradation was observed when purified EspPα was added to a growing culture of an EHEC-Hly-expressing strain, with isolated proteins and with coexpressing strains, and was independent of the EHEC serotype. EHEC-Hly breakdown occurred as a multistage process with the formation of characteristic fragments with relative molecular masses of ∼82 kDa and/or ∼84 kDa and ∼34 kDa. The initial cleavage occurred in the N-terminal hydrophobic domain of EHEC-Hly between Leu^235^ and Ser^236^ and abolished its haemolytic activity. In a cellular infection system, the cytolytic potential of EHEC-Hly-secreting recombinant strains was abolished when EspPα was coexpressed. EHEC in contact with human intestinal epithelial cells simultaneously upregulated their EHEC-Hly and EspP indicating that both molecules might interact under physiological conditions. We propose the concept of bacterial effector molecule interference (BEMI), reflecting the concerted interplay of virulence factors. Interference between effector molecules might be an additional way to regulate virulence functions and increases the complexity of monomolecular phenotypes.

## Introduction

Enterohaemorrhagic *Escherichia coli* (EHEC) cause diarrhoea, haemorrhagic colitis and the haemolytic uraemic syndrome (HUS) in humans (Karch *et al*., 2005; Tarr *et al*., 2005). In addition to *E. coli* O157 : H7, which is the most prevalent EHEC serotype worldwide (Banatvala *et al*., 2001; Tarr *et al*., 2005), a circumscribed panel of non-O157 : H7 EHEC serotypes have also been isolated from patients (Jelacic *et al*., 2003; Tozzi *et al*., 2003; Sonntag *et al*., 2004; Brooks *et al*., 2005; Karch *et al*., 2005; Johnson *et al*., 2006; Bielaszewska *et al*., 2007; Zhang *et al*., 2007; Hedican *et al*., 2009), the most common of which is O26 : H11/NM (non-motile) (Tozzi *et al*., 2003; Bielaszewska *et al*., 2007; Mellmann *et al*., 2008; Hedican *et al*., 2009).

Shiga toxins (Stx), the ribosome-inactivating AB_5_ proteins with rRNA *N*-glycosidase activity (Sandvig, 2001), are regarded as the major virulence factors of EHEC responsible for microvascular endothelial damage which underlies the pathogenesis of HUS (Bielaszewska and Karch, 2005; Tarr *et al*., 2005; Müthing *et al*., 2009). However, additional EHEC factors might also contribute to the pathogenesis of EHEC-mediated diseases (Brunder *et al*., 1997; Paton *et al*., 2004; Bielaszewska *et al*., 2005; Aldick *et al*., 2007; Gyles, 2007). Two such candidates are the serine protease EspP (Brunder *et al*., 1997) and the EHEC haemolysin (EHEC-Hly) (Schmidt *et al*., 1995; Aldick *et al*., 2007). EspP, a serine protease autotransporter of *Enterobacteriaceae* (SPATE), is among the most abundant secreted proteins of EHEC (Henderson and Nataro, 2001). This protein interacts with the coagulation cascade by cleaving factor V (Brunder *et al*., 1997) and with the complement system by degrading C3 and C5 (Orth *et al*., 2010). Besides this, a recent publication indicates that EspP might be involved in biofilm formation (Xicohtencatl-Cortes *et al*., 2010). The four recently identified EspP subtypes (α, β, γ and δ) differ in their transport and proteolytic capacities (Brockmeyer *et al*., 2007); notably, the proteolytically active EspPα is produced byEHEC associated with severe human disease (Brockmeyer *et al*., 2007; Khan *et al*., 2009). EHEC-Hly is a pore-forming cytolysin, which belongs to the RTX (repeat-in-toxin) family (Schmidt *et al*., 1995 and 1996). It lyses erythrocytes from different species and bovine lymphocytes (Schmidt *et al*., 1995; Bauer and Welch, 1996). Moreover, EHEC-Hly injures microvascular endothelial cells, suggesting a possible role in the pathogenesis of HUS (Aldick *et al*., 2007). Recently, we showed that EHEC-Hly are associated with outer membrane vesicles (OMVs) shed by EHEC. This mediates an up to 80 times increased stability and prolonged activity of the toxin as compared with its free, vesicle-unbound form (Aldick *et al*., 2009).

It has been shown that the interaction with bacterial proteases modulates biological activity of other members of the RTX family (Nagamune *et al*., 1996). We observed that EHEC strains of different serotypes harbouring the structural gene for EHEC-Hly differ by the presence and amount of EHEC-Hly in culture supernatants. To gain deeper insight into this phenomenon, we sought potential interaction partners of EHEC-Hly among the EHEC virulence factors. Here, we demonstrate that EspPα degrades and inactivates EHEC-Hly.

## Results

### Different expression of EHEC-Hly in wild-type EHEC strains

We observed substantial differences between EHEC strains in the amount of EHEC-Hly present in culture supernatants. Specifically, we detected full-length EHEC-Hly via immunoblot in supernatants of several EHEC serotypes, namely O6 : HNT, ONT : H25, O22 : H8, O84 : HNT, O113 : H21, O156 : NM and O163 : H19, but not EHEC O157 : H7 or O26 : H11/NM (Table 1). Interestingly, all strains containing detectable amounts of EHEC-Hly in supernatants harboured *espP*β, encoding the non-proteolytic EspPβ subtype, whereas the strains lacking EHEC-Hly in culture supernatants harboured *espP*α, encoding the highly proteolytic EspPα (Brockmeyer *et al*., 2007) (Table 1). We therefore hypothesized that the observed differences in the amount of EHEC-Hly present in these culture supernatants might be caused by an interaction of EHEC-Hly with EspPα, in particular that EspPα cleaves and thereby degrades EHEC-Hly. To test this hypothesis, we used recombinant EHEC-Hly and/or EspPα in order to eliminate the effect of other wild-type EHEC-secreted molecules that could influence their interactions.

**Table 1:** Differences in the amount of EHEC-Hly in culture supernatants of EHEC strains of different serotypes

Serotype^a^	No. of strains	*espP* subtype	EHEC*-hlyA*	EHEC-Hly in supernatant^b^
O26 : H11/NM	5	α	+	−
O157 : H7	1	α	+	−
O6 : HNT	1	β	+	+++
ONT : H25	1	β	+	++
O22 : H8	1	β	+	+
O84 : HNT	2	β	+	+
O113 : H21	1	β	+	+
O156 : NM	1	β	+	+
O163 : H19	1	β	+	+

a NM, non-motile strains; ONT, O antigen non-typeable; HNT, H antigen non-typeable.

b Determined by immunoblot of TCA-precipitated supernatants with anti-EHEC-Hly antibody; the intensities of the signals were determined using densitometry and classified as not detectable (−), detectable (+), abundant (++) and highly abundant (+++).

### Serine protease EspPα cleaves EHEC-Hly

To investigate possible interactions of EHEC-Hly with EspPα, we combined the two toxins with each other as isolated proteins, expressed them simultaneously or supplemented cultures containing EHEC-Hly with EspPα. We also studied if serotype-specific differences influence the interaction. Experimental conditions are summarized in Table 2 and strain constructs are described in Table 3.

**Table 2:** Overview of the experimental conditions used to assess interaction of EspPα and EHEC-Hly from EHEC O157 : H7 and O26 : H11.^a^

			(I) Proteolytic cleavage		(II) Haemolytic activity	
				
Experimental conditions			EspPα O157 : H7		EspPα O157 : H7	
		
Panel	EHEC-Hly		Isolated protein	Coexpression	Isolated protein	Coexpression
A	Growing culture	O157 : H7	X	n.a.^b^	X	n.a.
		O26 : H11	X	n.a.	X	n.a.
B	Isolated protein	O157 : H7	X	n.a.	X^c^	n.a.
		O26 : H11	X	n.a.	X^c^,^d^	n.a.
C	Coexpression	O157 : H7	n.a.	X	n.a.	X
		O26 : H11	n.a.	X	n.a.	X
D	OMVs	O157 : H7		n.a.		n.a.
		O26 : H11	X	n.a.	X	n.a.

a X indicates each particular set of experimental conditions used for studying the interaction of EspPα and EHEC-Hly.

b n.a.: not applicable due to experimental conditions.

c Freshly prepared culture supernatants of clones TA48 and TA50 were used in this experiment to ensure full haemolytic activity of EHEC-Hly.

d In addition, EspPα from O26 : H11 was used to perform the respective experiment.

**Table 3:** Description of recombinant clones used in this study

Construct	Description	Reference
pB 9-5	*espP* from EDL 933 (O157 : H7) transformed into *E. coli* DH5α (plasmid pB 9-5)	Brunder *et al*. (1997)
S263A	Inactive site-directed *espP* mutant in pB9-5 in *E. coli* DH5α (plasmid pS263A)	Brockmeyer *et al*. (2007)
TA48	EHEC-*hly* from EDL 933 (O157 : H7) in *E. coli* MC 1061 (plasmid pO157_EHEC-Hly_)	This study
TA50	EHEC-*hly* from 5157/96 (O26 : H11) in *E. coli* MC 1061 (plasmid pO26_EHEC-Hly_)	This study
TA142	Co-transformation of pO26_EHEC-Hly_ and pS263A in *E. coli* MC 1061	This study
TA143	Co-transformation of pO26_EHEC-Hly_ and pB 9-5 in *E. coli* MC 1061	This study
TA144	Co-transformation of pO157_EHEC-Hly_ and pS263A in *E. coli* MC 1061	This study
TA145	Co-transformation of pO157_EHEC-Hly_ and pB 9-5 in *E. coli* MC 1061	This study

**Fig. 1:**
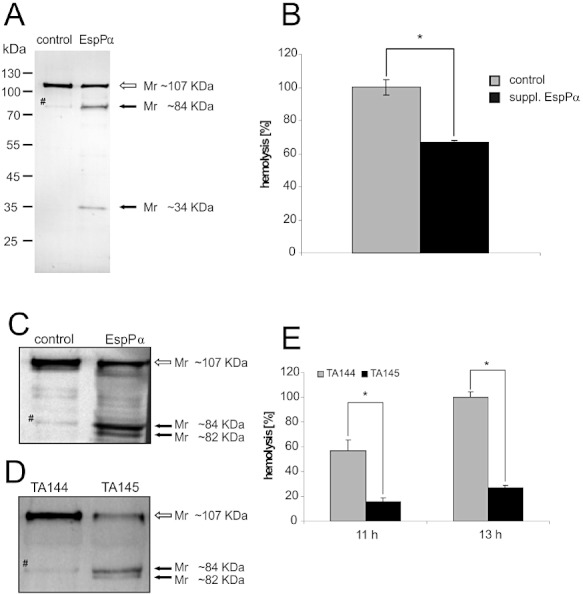
A and B. EspPα cleaves EHEC-Hly in bacterial culture. EHEC-Hly-producing strain TA48 was grown to early log phase and supplemented either with EspPα or with the EspPα-buffer control and incubated for further 2 h. (A) Sterile supernatants were TCA-precipitated, separated in SDS-PAGE and analysed in immunoblot using anti-EHEC-Hly antibody. The arrows indicate the 107 kDa band of EHEC-Hly (white arrow) or the specific Mr ∼84 kDa and ∼34 kDa breakdown fragments of EHEC-Hly (black arrows). The very weak immunoreactive band with a slightly higher Mr than that of the ∼84 kDa specific cleavage product (#) was present in all EHEC-Hly control preparations (see also C and D) and was therefore considered a background signal. (B) The sterile culture supernatants were assayed for their haemolytic activity, which was calculated as percentage of haemolysis (see *Experimental procedures*). Data in (B) are presented as means ± standard deviations of three independent assays. Statistically significant differences (*P* < 0.01, Student's *t*-test) are indicated by asterisk. C–E. Cleavage and inactivation of recombinant EHEC-Hly from EHEC O157 via EspPα from EHEC O157. Immunoblot analysis using anti-EHEC-Hly antibody of (C) recombinant isolated EHEC-Hly after incubation with buffer (control) or with recombinant purified EspPα, and (D) TCA-precipitated supernatants of clones TA144 and TA145 coexpressing recombinant EHEC-Hly and either EspPα (TA145) or the non-proteolytic EspPα mutant S263A (TA144). The arrows indicate the 107 kDa band of intact EHEC-Hly (white arrow) and the Mr ∼84 kDa and the ∼82 kDa EHEC-Hly cleavage products (black arrows). (E) Haemolytic activity of sterile culture supernatants of clones TA144 and TA145 after 11 h and 13 h of growth calculated as percentage of haemolysis. Data are presented as means ± standard deviations of at least three independent assays. Statistically significant differences between haemolytic activity of TA144 and TA145 (*P* < 0.01, Student's *t*-test) are indicated by asterisks.

In a first approach, we supplemented an early log-phase culture of clone TA48 producing recombinant EHEC-Hly from EHEC O157 : H7 with 5 µg ml^−1^ purified, recombinant EspPα from EHEC O157 : H7 or with an EspPα-buffer control (Table 2, panel A-O157 : H7-I) and continued incubation at 37°C for 2 h. Immunoblot analysis of trichloroacetic acid (TCA)-precipitated sterile supernatants using anti-EHEC-Hly polyclonal antibody demonstrated the occurrence of two immunoreactive breakdown products with relative molecular masses (Mr) of 84 ± 4 kDa and 34 ± 3 kDa in TA48 treated with EspPα (Fig. 1A, lane 2). These fragments were not present in TA48 supernatant treated with EspPα-buffer control (Fig. 1A, lane 1), indicating proteolytic cleavage of the 107 kDa large EHEC-Hly by EspPα. EspPα remained unaffected by EHEC-Hly as determined by immunoblot using an anti-EspP antibody. In addition, EspPα did not cross-react with the anti-EHEC-Hly antibody (data not shown).

To study the EspPα-mediated proteolytic cleavage of EHEC-Hly in more detail, we moved to the level of isolated recombinant proteins. Ammonium sulfate-precipitated EHEC-Hly from clone TA48 was incubated with purified, recombinant EspPα for 4 h at 37°C and analysed by immunoblot using anti-EHEC-Hly antibody (Table 2, panel B-O157 : H7-I). EHEC-Hly incubated with EspPα displayed two immunoreactive breakdown products of Mr ∼84 kDa and ∼82 kDa (Fig. 1C, lane 2). The Mr ∼34 kDa breakdown product was not observed at this timepoint (data not shown). Notably, prolonged incubation of EHEC-Hly with EspPα (> 18 h) led to the loss of the EHEC-Hly degradation bands. This indicated that after the initial formation of the Mr ∼84 kDa and ∼82 kDa breakdown products, there was further degradation (at least of the immunoreactive portion). Again, EspPα remained unaffected during the incubation with EHEC-Hly confirming the former observation.

We further sought to determine if the interaction of EspPα with EHEC-Hly is limited to isolated proteins or if it also occurs in cultures that coexpress both proteins (Table 2, panel C-O157 : H7-I). To this aim, sterile TCA-precipitated supernatants of 11 h and 13 h cultures of strains TA144 and TA145 were analysed by immunoblot using EHEC-Hly and EspPα antibodies. The supernatant of clone TA145, where proteolytically active EspPα was coexpressed with EHEC-Hly displayed, besides the 107 kDa band of the intact EHEC-Hly, again two breakdown products of Mr ∼84 kDa and ∼82 kDa (Fig. 1D, lane 2), while the Mr ∼34 kDa fragment was not observed at this time point. This is in accordance with the results observed for coincubated recombinant proteins (Fig. 1C). The specific breakdown products were not present in supernatant of clone TA144, in which the non-proteolytic EspPα mutant S263A (see *Experimental procedures*) was secreted simultaneously with EHEC-Hly (Fig. 1D, lane 1). Together, these data further affirm that the immunoreactive breakdown products resulted from the cleavage of EHEC-Hly by proteolytically active EspPα. The expression and secretion of EspPα and the non-proteolytic EspPα mutant in constructs TA145 and TA144, respectively, were confirmed by immunoblotting.

### EspPα cleavage cuts off haemolytic activity of EHEC-Hly

**Fig. 2:**
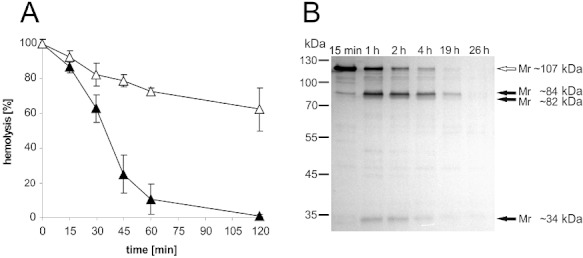
A. Haemolytic activity of EHEC-Hly is cut off by EspPα cleavage. EHEC-Hly-containing sterile culture supernatant from strain TA48 was incubated for indicated time intervals (0–120 min) with EspPα (▴) or EspPα-buffer control (▵) at 37°C and residual haemolytic activity was determined and calculated as percentage of haemolysis as described in *Experimental procedures*. Data are presented as means ± standard deviations of three independent assays. B. EHEC-Hly associated with OMV is cleaved by EspPα. Immunoblot analysis of OMV-associated EHEC-Hly incubated with EspPα for 15 min to 26 h using anti-EHEC-Hly antibody. The white arrow indicates the 107 kDa band of intact EHEC-Hly, and the black arrows mark the Mr ∼84 kDa, the ∼82 kDa and the ∼34 kDa EHEC-Hly cleavage products. Incubation times of OMV-associated EHEC-Hly with EspPα are given at the top of the figure.

To assess the functional consequences of EspPα-mediated cleavage for the biological activity of EHEC-Hly, we analysed the haemolytic activity of the sterile culture supernatant of EHEC-Hly-producing clone TA48 after the growing culture was supplemented with EspPα for 2 h (Table 2, panel A-O157 : H7-II). The EspPα treatment reduced the haemolytic activity of supernatant TA48 to 67%, compared to the EspPα-buffer control-treated sample (Fig. 1B), indicating that cleavage of EHEC-Hly eliminates its haemolytic activity. To further confirm this result, we tested culture supernatants of clones coexpressing EHEC-Hly and either EspPα (TA145) or the non-proteolytic mutant EspPα (TA144) for their haemolytic activities (Table 2, panel C-O157 : H7-II). Clone TA144 produced at different time points (11 h and 13 h) approximately fourfold higher haemolysis (57% versus 16% and 100% versus 27% respectively) than clone TA145 (Fig. 1E), demonstrating that the cleavage of EHEC-Hly by EspPα results in a significantly (*P* < 0.01) reduced haemolytic activity. In addition, sterile supernatant of clone TA48 containing EHEC-Hly was supplemented with 4 µg ml^−1^ recombinant EspPα or the respective buffer control, incubated at 37°C for different time intervals (0–120 min) and then tested for residual haemolytic activity (Table 2, panel B-O157 : H7-II). The activity of the EspPα-treated sample was reduced to 50% after about 35 min and totally abolished (1%) after 2 h as compared to its initial activity (Fig. 2A). The EHEC-Hly-containing supernatant exposed to the EspPα-buffer control also showed a reduction of haemolytic activity (from 100% to 62%) over time (Fig. 2A). This loss of activity in the control sample is due to the relatively short half-life time of the free EHEC-Hly itself, as reported previously (Aldick *et al*., 2009), the phenomenon that is thought to be caused by the irreversible self-aggregation of the toxin. Taken together, the rapid and complete loss of haemolytic activity of the EspPα-treated EHEC-Hly further underlines the capacity of EspPα to abolish the biological activity of EHEC-Hly by endoproteolytic cleavage.

### Analysis of EHEC-Hly breakdown fragments using mass spectrometry

**Fig. 3:**
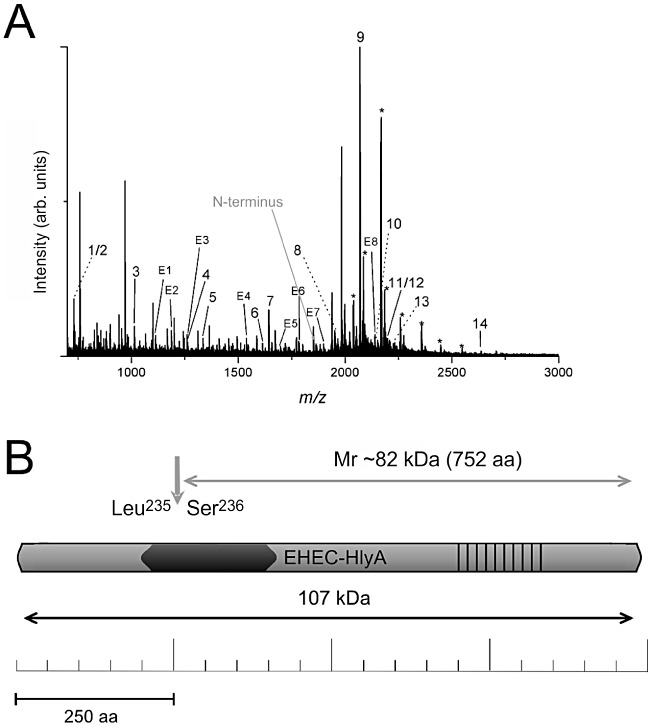
Mass spectrometric analysis of the trypsin-digested Mr ∼82 kDa breakdown fragment. A. Representative MALDI-TOF-MS spectrum of the Mr ∼82 kDa breakdown fragment of EHEC-Hly. Peaks identified as EHEC-Hly-specific (1–14), EspPα-specific (E1–E8) or EHEC-Hly-specific matrix adducts of 189 *m/z* units (*) are indicated. B. Schematic illustration of the 107 kDa large EHEC-Hly with its structural domains and the proposed cleavage site. The calcium-binding domain is indicated by strips and the hydrophobic domain is marked in dark grey. The EspPα cleavage site is located in the N-terminal end of the hydrophobic domain of EHEC-Hly as indicated by an arrow.

To verify the identity of the EHEC-Hly cleavage products and to estimate which domains might be affected, we analysed the immunoreactive breakdown fragments (Mr ∼84 kDa, ∼82 kDa and ∼34 kDa) by peptide mass fingerprinting using matrix-assisted laser desorption ionization time-of-flight mass spectrometry (MALDI-TOF-MS). In all of the three breakdown products, EHEC-Hly-specific peptides located in the C-terminal end of the protein were identified, indicating that after the formation of the Mr ∼84 kDa fragment further degradation leads to the generation of the smaller EHEC-Hly breakdown products. As an example, a representative peptide mass spectrum of the Mr ∼82 kDa fragment after tryptic digest is shown in Fig. 3A. In this EHEC-Hly fragment we identified 14 EHEC-Hly-specific peptides ranging in length between 6 and 24 amino acids resulting in a sequence coverage of 14.5% (Table 4, 1–14). The detected EHEC-Hly-specific peptides were located between amino acids 255 and 939 of the 998-amino-acid-large EHEC-Hly molecule (Fig. 3B). Several fragments were chemically modified because of reactions during sample preparation or data acquisition or formed matrix adducts (indicated in Table 4). The potential region of proteolytic cleavage by EspPα was determined to be located in the N-terminal end of EHEC-Hly as indicated by the arrow in the schematic illustration of the toxin (Fig. 3B). This is further underlined by the theoretical mass of 80.3 kDa of the EHEC-Hly fragment ranging from amino acid 255 to 998, which corresponds well to the observed migration pattern in gel electrophoresis (Fig. 1A). Further analysis of non-EHEC-Hly signals revealed the presence of eight peptides specific for EspPα (Table 4, peptides E1–E8). These peptides are entirely located in the N-terminal end of the serine protease and represent the previously described autodegradation product of EspPα (Dutta *et al*., 2002). This EspPα-specific fragment, which is commonly found in all EspPα isolations, has a Mr of ∼86 kDa and accompanied also the isolated Mr ∼82 kDa breakdown product of EHEC-Hly due to a similar electrophoretic separation property as evidenced by immunoblotting analysis (data not shown).

**Table 4:** Tryptic peptides identified in the ∼82 kDa EHEC-Hly breakdown product using MALDI-TOF-MS

Peak^a^	*m/z*_theor._[M+H]^+^	*m/z*_exp._[M+H]^+^	Position	Modification	Missed cleavages	Sequence
1	731.4	731.3	921–926		1	2EIKVDK
2	735.4	735.4	653–658		0	DTWSVK
3	1011.5	1011.5	321–328		0	QLESYSER
4	1264.6	1264.6	411–420	Deamidated	1	FAARINEWEK
5	1335.7	1335.7	924–934		1	VDKIPHNNNER
6	1613.9	1613.8	319–331		3	AKQLESYSERFKK
7	1641.9	1641.8	255–271		0	AAAGIELTTQVLGNVGK
8	1950.0	1949.9	668–683		3	EQEVSVGKRTEKIQYR
9	2069.1	2069.0	572–588	Deamidated	2	NHKGVYDYSKLIQFVEK
10	2151.0	2151.0	551–568	Deamidated, oxidized	1	YEYMTSLIVNGKDTWSVK
11	2226.1	2226.0	563–581	Deamidated	3	DTWSVKGIKNHKGVYDYSK
12	2232.1	2232.0	544–562	Deamidated	3	ERKQSGKYEYMTSLIVNGK
13	2255.2	2255.0	921–939	Deamidated	3	EIKVDKIPHNNNERSGYIK
14	2652.3	2652.2	654–676	Deamidated, oxidized	2	NMYGDVEVLQEVVKEQEVSVGKR
N-term	1862.9	1862.9	236–254		0	SAVSASFILGNSDAHTGTK
E1	1113.6	1113.7	745–755		0	ASVVGDIHSTK
E2	1185.6	1185.7	134–143		2	YGVNYKGEKK
E3	1259.7	1259.8	149–161	Deamidated	0	AGSGVVSVKKNGR
E4	1546.8	1546.9	668–680	Deamidated	0	ALFSNYVYLLNTK
E5	1696.8	1696.8	339–352	Deamidated	1	NGSTVEWNIRYDNK
E6	1783.9	1783.9	128–142	Deamidated	2	QQALERYGVNYKGE
E7	1899.9	1899.9	222–238	2 Deamidated	0	WVVAGTVWGIYNYANGK
E8	2138.0	2138.0	562–580		0	TNNAVSDLSQPDWETGTFR

a Mass signals derived from EHEC-Hly are numbered from 1 to 14, and signals from the EspPα autodegradation fragment are marked as E1 to E8. N-term is the corresponding mass signal to the newly formed N-terminus of the EHEC-Hly breakdown product.

Attempts to identify the potential EHEC-Hly cleavage site by N-terminal sequencing were unsuccessful, probably because of comigrating EspPα autodegradation products, leading to ambiguous sequencing results. Therefore, we employed two overlapping synthetic peptides covering the EHEC-Hly sequence from residue 191 to 250 and incubated each peptide overnight with EspPα and S263A as a negative control. The formation of peptide fragments by EspPα-mediated cleavage was analysed by MALDI-TOF-MS and displayed a specific mass signal at *m/z* 1475.7 corresponding to a peptide fragment where EspPα cleavage would occur after Leu^235^. Re-examination of peptide mass fingerprinting data of the Mr ∼82 kDa fragment showed, in addition, a signal at *m/z* 1862.9 which corresponds to residues Ser^236^ to Lys^254^; this further supports the above data that cleavage occurs after Leu^235^ resulting in a newly formed N-terminus at Ser^236^ (Fig. 3 and Table 4).

### EspPα from EHEC O26 : H11 cleaves and inactivates EHEC-Hly from EHEC O26 : H11

To assess whether or not the cleavage and inactivation of EHEC-Hly by EspPα is limited to serotype O157 : H7, we isolated both toxins from EHEC of serotype O26 : H11. EHEC-Hly_O26_ (originating from clone TA50) and EspPα_O26_ (originating from wild-type EHEC O26 : H11 strain 5631/96) were coincubated for 4 h and the mixture was assayed for haemolytic activity. Similar to observations for the proteins from EHEC O157 : H7, EHEC-Hly_O26_ was inactivated by EspPα_O26_ as demonstrated by loss of its haemolytic activity (data not shown). Hence, cleavage and inactivation of EHEC-Hly by EspPα is not restricted to the O157 : H7 serotype.

### Interaction of EspPα from EHEC O157 : H7 and EHEC-Hly from EHEC O26 : H11

To determine if cleavage of EHEC-Hly by EspPα is limited to proteins derived from the same serotype (i.e. O157 : H7 or O26 : H11) or if this effect is also observed between proteins from different serotypes, we investigated the interaction of EspPα from EHEC O157 : H7 with EHEC-Hly from EHEC O26 : H11. Similar to the above experiments, the interaction was studied by supplementation of growing cultures containing EHEC-Hly with purified EspPα (Table 2, panel A-O26 : H11-I and II), co-incubation of isolated recombinant proteins (Table 2, panel B-O26 : H11-I and II) and coexpression of recombinant plasmids using clones TA143 (expressing EHEC-Hly and EspPα) and TA142 (expressing EHEC-Hly and the mutant, non-proteolytic, EspPα) (Table 2, panel C-O26 : H11-I and II). These experiments demonstrated that EHEC-Hly from EHEC O26 : H11 is cleaved and its haemolytic activity is abolished via EspPα from EHEC O157 : H7 (Fig. S1). The results were comparable to those shown above for the respective proteins from the same serotype (Fig. 1A–C). This indicates that the cleavage and functional inactivation of EHEC-Hly via EspPα is serotype-independent and suggests a general principle underlying the interaction of these two proteins.

### EspPα cleaves and inactivates EHEC-Hly associated with OMVs

Recently, we reported that EHEC-Hly secreted by EHEC rapidly binds to OMVs shed by the bacteria and that this OMV association stabilizes the toxin and significantly prolongs its haemolytic activity (Aldick *et al*., 2009). This prompted us to investigate whether OMV-associated EHEC-Hly might be protected from cleavage by EspPα. OMVs carrying EHEC-Hly freshly isolated from strain TA50 were incubated with 2 µg ml^−1^ EspPα for different time intervals (15 min to 26 h) at 37°C and subsequently analysed in immunoblot using anti-EHEC-Hly antibody (Table 2, panel D-O26 : H11-I). Notably, already 15 min after exposure to EspPα the characteristic Mr ∼84 kDa immunoreactive breakdown product appeared and remained visible up to 19 h, while the band corresponding to the intact OMV-associated EHEC-Hly steadily diminished (Fig. 2B). The fragments of Mr ∼34 kDa and ∼82 kDa, the latter of which remained less defined, were present after 1 h of incubation and diminished over time. After > 19 h, all four immunoreactive bands completely disappeared (Fig. 2B). This indicates that the association with OMV does not protect EHEC-Hly from proteolysis by EspPα.

In accordance with these immunoblot data, exposure to EspPα for 20 h (Table 2, panel D-O26 : H11-II) completely ablated the haemolytic activity of OMV-associated EHEC-Hly.

Outer membrane vesicle-associated EHEC-Hly causes haemolysis first after 15 h of incubation with erythrocytes probably because of the necessary maturation process of vesicles (Aldick *et al*., 2009). Because EspPα rapidly cleaves OMV-associated EHEC-Hly (within 4 h) (Fig. 2B), complete absence of haemolytic activity of EspPα-treated OMV-associated EHEC-Hly results from degradation of EHEC-Hly before vesicle maturation.

### Chronology of EHEC-*hlyA* and *espP* expression

To determine if EHEC-Hly and EspP could theoretically come into direct contact during infection, we studied the chronology of EHEC-*hlyA* and *espP* expression in four selected EHEC strains producing EHEC-Hly together with EspPα (O157 : H7 strain EDL933 and O26 : H11 strain 5236/96) or EspPβ (O6 : HNT strain 3503/98 and O163 : H19 strain 36/03; Table 1) under laboratory and *in vivo* mimicking conditions. All four strains were cultured in LB broth under standard laboratory conditions (37°C, rotary shaker) for 2 h, 4 h, 8 h, 12 h, 16 h, 20 h and 24 h, respectively, and mRNA transcription level of EHEC-*hlyA* and *espP* were determined relative to the housekeeping gene *gapA* (used as a reference) in quantitative reverse transcription (RT)-PCR. The strains showed in general slightly elevated expression of both EHEC-*hlyA* and *espP* with an increase of up to threefold relative to the 2 h time point (Fig. S2), except for strain 5236/96 where EHEC-*hlyA* expression was not increased. Upregulation peaked at the high cell density (> 16 h); in strain 3503/98 (O6 : HNT) the *espP* expression was increased up to eightfold. No significant difference was observed between strains expressing proteolytically active EspPα (EDL933, 5236/96) or inactive EspPβ (3503/98, 36/03).

**Fig. 4:**
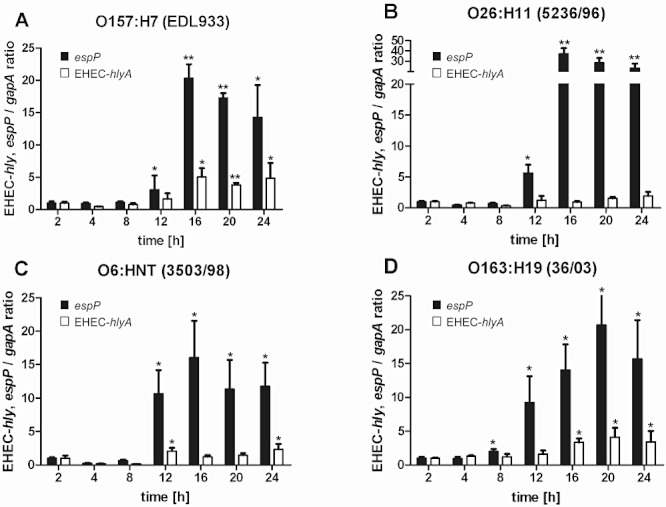
Chronology of EHEC-*hlyA* and *espP* expression in EHEC strains during contact with human intestinal epithelial cells. HCT-8 monolayers were infected with overnight cultures of EHEC strains producing EHEC-Hly together with either EspPα (O157 : H7 strain EDL933 and O26 : H11 strain 5236/96) (A and B) or EspPβ (O6 : HNT strain 3503/98 and O163 : H19 strain 36/03) (C and D) for 2–24 h as indicated. Bacteria were harvested by centrifugation, RNA was isolated and transcription levels of EHEC-*hlyA* and *espP* were determined using RT-PCR and normalized to *gapA*. Upregulation of each gene expression relative to 2 h time point was determined using Student's *t*-test with *P < 0.05 and **P < 0.001. Data are means ± standard deviations from two independent assays.

To investigate the expression of both virulence factors under conditions mimicking the situation during human infection, we developed an intestinal cell infection assay. Human ileo-caecal epithelial cells (HCT-8) were infected with overnight cultures of the same set of EHEC strains tested above and incubated at 37°C under static conditions for 2 h, 4 h, 8 h, 12 h, 16 h, 20 h and 24 h. Relative transcription of EHEC-*hlyA* and *espP* mRNA was again normalized to *gapA* (Fig. 4). In the presence of intestinal epithelial cells, expression of both EHEC-*hlyA* and *espP* was significantly upregulated compared with LB media. After 12 h, EHEC-*hlyA* expression increased by two- to fivefold relative to the starting conditions (2 h time point). Similarly, *espP* was significantly upregulated after 12 h leading to 15- to 20-fold increase; in strain 5236/96 (O26 : H11) upregulation reached even > 35-fold (Fig. 4). Again, no significant difference between strains expressing proteolytically active EspPα and inactive EspPβ was observed.

Together, these data demonstrate that EHEC-*hlyA* and *espP* are expressed simultaneously both under laboratory conditions and under *in vivo* mimicking conditions, suggesting that EHEC-Hly and EspP could come together during infection. The more pronounced and simultaneous upregulation of both virulence factors after contact of EHEC with intestinal epithelial cells, the first barrier encountered by the organisms during human disease, indicates that both molecules may be effectors during infection.

### Biological consequence of EHEC-Hly cleavage by EspP

**Fig. 5:**
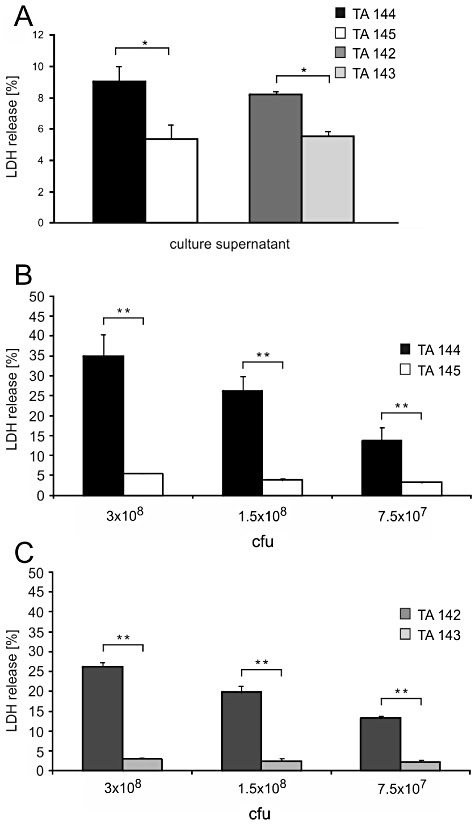
Consequences of EHEC-Hly cleavage by EspPα on cytolytic activity of EHEC-Hly. HBMEC monolayers were exposed to supernatants of overnight cultures of clones TA145, TA144, TA143 and TA142 which produce EHEC-Hly together with proteolytically active EspPα (TA145, TA143) or proteolytically inactive EspPα mutant S263A (TA144, TA142) (A) or to decreasing doses (3 × 10^8^ to 7.5 × 10^7^ cfu) of bacterial cultures of the respective clones (B and C) as described in *Experimental procedures*. HBMEC lysis was monitored by measuring release of intracellular LDH using the CytoTox 96 kit. The differences between HBMEC lysis caused by clones expressing proteolytically active EspPα and the non-proteolytic S263A were determined using Student's *t*-test with *P < 0.05 and **P < 0.001. Data are means ± standard deviations from four measurements.

To investigate if the cleavage of EHEC-Hly by EspP might have biological implications during an EHEC infection, we used a model of human brain microvascular endothelial cells (HBMECs). The brain microvasculature is a target during EHEC-mediated HUS and these cells are highly susceptible to the cytolytic effect of EHEC-Hly (Aldick *et al*., 2007). Cultured HBMECs were first exposed to supernatants of overnight cultures of clones TA145/TA144 and TA143/TA142 producing EHEC-Hly from EHEC O157 and EHEC O26, respectively, together with proteolytically active/inactive EspPα, and HBMEC lysis was monitored by measuring release of intracellular lactate dehydrogenase (LDH). In both pairs, the clones expressing EHEC-Hly together with inactive EspPα (TA144 and TA142) caused lysis of HBMECs which was low, but significantly higher than that caused by the clones that express EHEC-Hly together with the proteolytically active EspPα (TA145 and TA143) (Fig. 5A). To enhance the effect of EHEC-Hly on the target cells, we next infected the HBMEC monolayers with living bacteria which continuously produce the toxin, increasing therefore the probability of its interaction with the cells. After 4 h exposure of HBMECs to the bacterial cultures and subsequent post-incubation for 18 h, clones TA144 and TA142 caused a pronounced dose-dependent lysis of HBMECs, whereas clones TA145 and TA143 caused only baseline and dose-independent LDH release (Fig. 5B and C). This suggests that cleavage of EHEC-Hly by EspPα might prevent the lytic effect of the toxin on the target cells.

## Discussion

Most studies of EHEC virulence factors have focused on isolated molecules such as Shiga toxins (Hurley *et al*., 2001; Lee *et al*., 2008), cytolethal distending toxin (Bielaszewska *et al*., 2005), subtilase toxin (Paton *et al*., 2004; Chong *et al*., 2008), EHEC-Hly (Bauer and Welch, 1996; Schmidt *et al*., 1996; Aldick *et al*., 2007; 2009) and EspP (Brunder *et al*., 1997). Although such an approach is a prerequisite for understanding exact mechanisms of action of particular virulence factors, its obvious drawback is that potential interactions between the various effector molecules produced by an EHEC strain are not considered. Experiments restricted to single toxins might thus lead to inadequate conclusions and rather simplified interpretations of the toxins' contributions to the biological effects observed during infection.

An example for multifactorial protein interactions is the activation of toxin precursors via proteolytic cleavage, which can be mediated either by host or by bacterial proteases. More specific, the eukaryotic cellular protease furin cleaves the Stx A subunit, generating a catalytically active A_1_ fragment with rRNA *N*-glycosidase activity (Garred *et al*., 1995). Similarly, cleavage of the last two amino acids from the A_2_ fragment of the A subunit of Stx2d via elastase in the intestinal mucus dramatically increases cytotoxicity of this toxin (Melton-Celsa *et al*., 2002; Bielaszewska *et al*., 2006). An example of a bacterial protease with a precursor-activating effect is the *Vibrio cholerae* haemagglutinin/protease which cleaves, and thereby activates, *V. cholerae* haemolysin, another member of the RTX family (Nagamune *et al*., 1996).

In contrast to these examples, the cleavage and inactivation of a bacterial toxin by a bacterial protease secreted from the same organism, has, to our knowledge, been demonstrated for the first time in the present study.

The data we present are likely to reflect a genuine biologic process, in view of the different situations in which we demonstrated this effect, including purified EspPα added to a strain expressing EHEC-Hly, effector molecules co-incubated as isolated proteins, and finally, the interaction of EspPα and EHEC-Hly coexpressed by recombinants. Regardless in which form both molecules are brought together, EHEC-Hly is degraded and functionally inactivated by EspPα. Furthermore, this effect is also observed when EspPα and EHEC-Hly are derived from different serotypes. The cleavage appears to be a multistage process in which, depending on the specific incubation time and conditions, breakdown products of different sizes are observed. The initial cleavage results in the formation of a Mr ∼82 kDa and/or an ∼84 kDa breakdown fragment and proceeds further to generate a Mr ∼34 kDa fragment (Figs 1 and 2B). The time-course of the formation of the Mr ∼84 kDa, ∼82 kDa and the ∼34 kDa fragment is well documented and exemplarily shown for the OMV-associated EHEC-Hly (Fig. 2B). Specifically, the appearance of the Mr ∼34 kDa band after 1 h and its strong reduction already after 4 h of exposure of EHEC-Hly to EspPα (Fig. 2B) may explain why this fragment was observed neither after 4 h of co-incubation of the recombinant proteins (Fig. 1C) nor in the supernatant of clone TA145 after overnight growth (Fig. 1D). Prolonged incubation with EspPα finally leads to a complete proteolytic degradation of OMV-associated EHEC-Hly.

Analysis using mass spectrometry confirmed the identity of the Mr ∼84 kDa, ∼82 kDa and ∼34 kDa breakdown products as EHEC-Hly-specific. Notably, the initial cleavage of EHEC-Hly occurs at position Leu^235^ in the early N-terminal end of the toxin. In this region a domain of predominantly hydrophobic amino acids is located (between amino acid 210 and 410) (Schmidt *et al*., 1995). This domain is proposed to be crucial for the biological activity of RTX toxins in general, as it is involved in the interaction with target cell membranes leading to pore formation (Welch, 2001). The cleavage of EHEC-Hly via EspPα in the region of the hydrophobic domain thus plausibly explains the immediate reduction of its haemolytic activity, as the functionality of this domain is most probably abolished. Interestingly, we were not able to detect any larger fragments of the N-terminal hydrophobic domain using MALDI-TOF-MS, indicating that this region might be degraded to smaller peptides. However, we cannot exclude the possibility that this region is not recognized by the employed polyclonal anti-EHEC-Hly antibody.

Currently, the physiological significance of the cleavage and inactivation of EHEC-Hly by EspPα remains unclear. Prolonged exposure of EHEC bacteria to EHEC-Hly might, for example, directly or indirectly cause detrimental effects to the producing bacteria and EspPα might serve as a specific inactivator for EHEC-Hly. Although we were not able to detect such direct antibacterial toxic effects under laboratory conditions, we cannot exclude the possibility that EHEC are affected by their own haemolysin in a physiological environment. An animal model of acute haemorrhagic colitis, ideally with systemic thrombotic microangiopathy, would be very useful in establishing the relevance of our findings to mammalian infection. However, the human host is a singular victim of EHEC infections from STEC of the serotypes studied, and a suitable model does not, regrettably, exist, for us to perform such experiments (Mundy *et al*., 2006). Our results using *in vivo* mimicking systems substantiate the possible relevance of the findings for the *in vivo* situation. Analysis of the EHEC-*hlyA* and *espP* expression demonstrated that both toxins are moderately upregulated especially at higher cell densities. Intriguingly, expression of both toxins was substantial increased when EHEC were grown in direct contact with cultured epithelial cells from human colon. This suggests that both toxins might be expressed during infection, in particular after EHEC colonize the large intestinal epithelium. Since EHEC-*hlyA* and *espP* were expressed simultaneously in all four tested strains, the two toxins could stand a realistic chance to physically interact during natural infection. Abolishment of the EHEC-Hly cytolytic activity towards the brain microvascular endothelium using recombinant strains coexpressing EspPα further demonstrates that EspPα may act as an inactivator for EHEC-Hly in cells that are affected during extra-intestinal complications of EHEC infection, such as HUS. Even though cell culture models have certain limitations, our results suggest that the observed cleavage and inactivation of EHEC-Hly might also take place *in vivo* during infection.

Although its significance during human infection is presently unknown, the cleavage and inactivation of EHEC-Hly via EspPα suggest that pathogens can regulate their virulence phenotypes by interference of effector molecules. Therefore, we propose the concept of bacterial effector molecule interference (BEMI), reflecting the concerted interplay of proven and potential virulence factors, thus increasing the complexity of monomolecular phenotypes. Further studies addressing in detail different aspects of the interaction of the virulence factors with their target cells (e.g. the way and the kinetics of the cellular delivery) are clearly required to fully understand the significance of the interaction of EHEC-Hly and EspP (and perhaps also other virulence factors) in the context of human infections.

## Experimental procedures

### Bacterial strains and plasmids

The following constructs were used in this study (Table 3): the plasmids pO157_EHEC-Hly_ (Schmidt *et al*., 1995) and pO26_EHEC-Hly_ (Aldick *et al*., 2007) harbouring the EHEC-*hly* operon from EHEC O157 : H7 strain EDL933 and EHEC O26 : H11 strain 5157/96, respectively, were transformed into *E. coli* K12 strain MC1061, resulting in clones TA48 and TA50 respectively. The plasmid pB 9-5 harbouring the open reading frame of *espP* from EHEC O157 : H7 EDL933 (Brunder *et al*., 1997) and the plasmid pS263A (Brockmeyer *et al*., 2009), obtained by site-directed mutagenesis (Site-directed mutagenesis Kit, Stratagene, La Jolla, CA, USA) of pB 9-5 at base pair position 789 leading to amino acid change of serine to alanine, were each transformed into *E. coli* K12 strain DH5α (clones pB 9-5 and S263A respectively) (Brockmeyer *et al*., 2009). Coexpressing clones were obtained by co-transformation of *E. coli* K12 strain MC1061 with pO26_EHEC-Hly_ together with either pS263A (TA142) or pB 9-5 (TA143) or with pO157_EHEC-Hly_ together with either pS263A (TA144) or pB 9-5 (TA145). Furthermore, a wild-type EHEC O26 : H11 from our collection (strain 5631/96) was used as a source of EspPα.

Fourteen EHEC isolates analysed for the presence of EHEC-Hly in culture supernatants are listed in Table 1. The presence of the structural gene for EHEC-Hly (EHEC-*hlyA*) and the *espP* subtypes in these strains (all isolated in our laboratory) were determined using PCR (Schmidt *et al*., 1995; Brockmeyer *et al*., 2007). Expression of EHEC-Hly was confirmed by the strains' ability to produce enterohaemolytic phenotype on enterohaemolysin agar (Schmidt *et al*., 1995). Production of EspP and its proteolytic activity were determined using an immunoblot and the ability of culture supernatants to cleave an oligopeptide substrate, respectively, as described previously (Brockmeyer *et al*., 2007).

### Antibodies

Rabbit polyclonal antibodies and their dilutions were used as follows: anti-EHEC-Hly 1:10 000 (Schmidt *et al*., 1995) and anti-EspP 1:1000 (Brunder *et al*., 1997). Alkaline phosphatase-conjugated goat anti-rabbit IgG (Jackson ImmunoResearch, Baltimore, MA, USA) diluted 1:10 000 was used as secondary antibody.

### Preparation of recombinant EHEC-Hly and EspPα

EHEC-Hly and EspPα were isolated and purified as described previously (Aldick *et al*., 2007; Brockmeyer *et al*., 2007). Briefly, EHEC-Hly-producing strains TA48 and TA50 encoding EHEC-Hly from EHEC O157 : H7 and O26 : H11, respectively, were grown overnight on enterohaemolysin agar (SIFIN, Berlin, Germany), inoculated into 150 ml of Luria–Bertani (LB) broth (supplemented with 100 µg ml^−1^ ampicillin) and incubated (13 h, 37°C, 180 r.p.m.). For experiments using early log-phase cultures, strains were grown (37°C, 180 r.p.m.) in 50 ml of LB broth (with 100 µg ml^−1^ ampicillin) for 6 h. Strains expressing EspPα, the site-directed mutant and the wild-type EHEC O26 : H11 strain 5631/96 were grown overnight in 50 ml of LB broth at 37°C with vigorous shaking. Sterile culture supernatants of all strains were used directly or proteins were precipitated (1 h, 4°C) by adding ammonium sulfate to 55% saturation. Precipitates were sedimented (5500 *g*, 30 min, 4°C) and dissolved in HEPES buffer (10 mM HEPES, 150 mM NaCl, pH 7.4). EspPα was purified using HiTrap Benzamidine FF columns (GE Healthcare, Munich, Germany) according to the manufacturer's instructions. The fractions enriched for EspPα were collected and concentrated using a 10 kDa Vivaspin spin-down filter (Vivascience-Sartorius, Göttingen, Germany). OMV-associated EHEC-Hly was isolated from strain TA50 using ultracentrifugation as described previously (Aldick *et al*., 2009). OMVs obtained from 300 ml of sterile culture supernatant were resuspended in 400 µl of HEPES buffer. This preparation was used to test both proteolytic cleavage (applying 10 µl of OMV preparation) and haemolytic activity (applying 100 µl of OMV preparation).

### Coexpression of EHEC-Hly and EspPα

Strains TA142, TA143, TA144 and TA145 were grown overnight on enterohaemolysin agar, inoculated into 150 ml of LB broth supplemented with 100 µg ml^−1^ ampicillin and 30 µg ml^−1^ kanamycin and incubated for 11 h and 13 h at 37°C and 180 r.p.m. Cell free supernatants were either used directly in haemolytic activity assay or 1 ml was precipitated using 10% TCA (1 h, 4°C), and the precipitate was sedimented (20 000 *g*, 30 min, 4°C), resuspended in 30 µl of sample buffer (20 mM Tris HCl, pH 8.0) and separated electrophoretically for immunodetection.

### Sodium dodecylsulfate polyacrylamide gel electrophoresis (SDS-PAGE) and immunoblotting analyses

Samples including TCA-precipitated supernatants (see above) of clones TA142, TA143, TA144 and TA145 or of wild-type EHEC strains (Table 1), and ammonium sulfate-precipitated EHEC-Hly treated with EspPα or with a buffer control were analysed using standard SDS-PAGE (Laemmli, 1970). Separated proteins were transferred to PVDF membrane (Carl Roth, Karlsruhe, Germany) using Trans-Blot SD (Bio-Rad, Munich, Germany) semi-dry blotting system (1 h, 50 mA). Different primary antibodies and a secondary antibody were used as listed above. Detection was achieved using the chromogenic substrates 5-Bromo-4-chloro-3′-indolylphosphate *p*-toluidine salt and *p*-Nitro-Blue tetrazolium chloride (both from Carl Roth) and signal intensities determined by densitometry (Quantity One®, Bio-Rad, Munich, Germany).

### Haemolysis assay

Haemolytic activity was quantified as described previously (Aldick *et al*., 2007), with slight modifications. Briefly, 950 µl of sterile culture supernatant of strain TA50 containing 5 mM CaCl_2_ was supplemented with 50 µl of an EspPα preparation (described above) and incubated at 37°C for indicated time points (0–120 min). Subsequently, 100 µl of an erythrocyte suspension (10% human erythrocytes washed thrice with PBS) was added and incubation was continued for 4 h at 37°C with gentle shaking. Erythrocytes were sedimented by centrifugation (400 *g*, 5 min) and absorbance of the clear supernatant was measured at OD_570_. A microtitre assay was used to quantify haemolytic activity of OMV-associated EHEC-Hly (Aldick *et al*., 2009). Briefly, 100 µl of OMV preparations were diluted twofold in the assay buffer (0.9% NaCl containing 10 mM CaCl_2_) in a 96-well plate and 50 µl of an erythrocyte suspension was added. The plate was incubated at 37°C with gentle shaking for 20 h, and the erythrocytes were sedimented by centrifugation (400 *g*, 5 min). Clear supernatants were transferred to a fresh plate and OD_570_ was measured. Either LB broth (for supernatants) or HEPES buffer (for OMVs) were used as a background and distilled water as a total lysis control. The percentage of haemolysis was calculated for both assays as follows: % haemolysis = (OD_570_ of sample − OD_570_ of background)/(OD_570_ of total − OD_570_ of background) × 100.

### Peptide mass fingerprinting using MALDI-TOF-MS

Samples containing the EHEC-Hly breakdown fragments were separated electrophoretically, transferred to a PVDF membrane and a stripe of the membrane was immunostained using anti-EHEC-Hly antibody. This served to identify the characteristic breakdown fragments. Electrophoretically separated proteins were incubated with 300 ng of sequencing grade trypsin (Roche, Mannheim, Germany) in 50 mM NH_4_HCO_3_ for 5 h at room temperature according to Shevchenko and colleagues (1996) with minor modifications (Müthing *et al*., 2004). Saturated α-cyano-4-hydroxycinnammic acid in 50% acetonitril and 0.1% trifluoracetate was applied to the digest as a matrix. MALDI-TOF-MS was performed with a prototype of a prOTOF 2000 mass spectrometer by Perkin Elmer (from Sciex, Concordia, Canada) in positive ion mode. A pulsed N_2_-laser (λ = 337 nm) was used for ultraviolet matrix-assisted laser desorption/ionization orthogonal time-of-flight (UV-MALDI-o-TOF) mass spectrometry. Identification of trypsin-digested peptides was performed by comparing molecular ions [M+H]^+^ (*m/z*_exp._) with ions of theoretically digested fragments of EHEC-Hly (*m/z*_theor._) using ProteinProspector (UCSF Proteomic tools, v4.27.2) and the ALDENTE peptide mass fingerprinting tool (available at the ExPASy proteomics server from the SWISS Institute of Bioinformatics SIB).

### Digestion of synthetic EHEC-Hly peptides

For a more detailed analysis of EHEC-Hly cleavage sites two overlapping synthetic peptides covering the EHEC-Hly sequence from residue 191 to 250 (sequence derived from EHEC O157 : H7 EDL 933) were custom synthesized (Selleck, Houston, Texas). Sequences of peptides were: Ehly1 (FSEQLNQLGS FLSSKPRLS SVGGKLQNLPD) (covering residue 191–220) and Ehly2 (QNLPDLGPLG DGLDVVSGIL SAVSASFILG NSDAH) representing residue 216–250. One hundred microlitres of a 2 mM solution of each peptide dissolved in PBS was incubated overnight with 2.5 µg of EspPα or the negative control S263A at 37°C overnight. Peptide solutions were desalted using C18 SPE cartridges (Phenomenex, Germany, Aschaffenburg) and stepwise elution. Mass spectrometric analysis was performed on a prOTOF 2000 mass spectrometer by Perkin Elmer (from Sciex, Concordia, Canada) in positive ion mode as described above.

### Chronology of EHEC-*hlyA* and *espP* expression

Chronology of the expression of EHEC-*hlyA* and *espP* in selected EHEC strains producing EHEC-Hly together with EspPα (O157 : H7 strain EDL933 and O26 : H11 strain 5236/96) or EspPβ (strains of serotypes O6 : HNT and O163 : H19; Table 1) was investigated using quantitative RT-PCR using two different experimental conditions. First, the strains were grown in LB broth at 180 r.p.m. and 37°C for 2 h, 4 h, 8 h, 12 h, 16 h, 20 h and 24 h. At each time interval bacteria were harvested by centrifugation and RNA was isolated as described below. Second, the expression of each toxin was analysed after contact of the bacteria with human intestinal epithelial cells (HCT-8) to simulate a situation during infection. HCT-8 cells (human ileo-caecal adenocarcinoma epithelial cells; ATCC CCL-244) were grown in RPMI 1640 (Lonza, Cologne, Germany) supplemented with 10% fetal calf serum (PAA, Pasching, Austria), 2 mM l-glutamine and 1 mM sodium pyruvate (Lonza). For the experiment, cells were seeded (2.5 × 10^5^ cells per well) into six-well plates (Corning, Corning, NY, USA) and grown until ∼70% confluence. Cells were washed three times with phosphate-buffered saline (PBS), infected with 2 ml (1 × 10^8^ cfu) of bacterial overnight cultures in LB broth and incubated with the bacteria (37°C, 5% CO_2_, 0.5% mannose in cell culture medium) for 2 h, 4 h, 8 h, 12 h, 16 h, 20 h and 24 h. To recover at each time point both bacteria that adhered to the cells during the incubation period and those that remained non-adherent, medium from the cells in each well was collected and the cells were harvested by trypsinization. Both the cell culture medium and trypsinized cells were centrifuged (2000 *g*, 10 min, 4°C), and pellets were pooled, washed twice with PBS and used for RNA extraction.

### Total RNA isolation

Total bacterial RNA was isolated using the RNeasy Mini Kit (Qiagen, Hilden, Germany) according to manufacturer's instruction. To eliminate contamination by genomic DNA, all RNA samples were treated with DNase I (Roche Diagnostics, Mannheim, Germany). The concentration of RNA was determined and its quality was checked by measuring optical density at 260 nm.

### Quantitative RT-PCR

A one-step quantitative RT-PCR, performed with an iCycler iQ-5 (Bio-Rad, Munich, Germany) and 2 × SensiMix™ SYBR One-Step Kit (Peqlab Biotechnologie, Erlangen, Germany), was used to measure the relative expression of EHEC-*hlyA* and *espP* mRNA. The PCR reactions were performed in a 96-well plate using a 20 µl volume containing 1 µl of total RNA (100 ng), 10 µl of 2 × SensiMix™ SYBR One-Step, 0.4 µl of RNase Inhibitor and 200 nM of each primer. Primers HlyA9 (5′-ACC CCA GGA GAA GAA GTT AG-3′) and HlyA4 (5′-TCT CGC CTG ATA GTG TTT GGT A-3′), EspP-RTF (5′-GCT CCA CCC TGA AAC TAC CG-3′) and EspP-RTR (5′-CGT TCA AGT GCC TGC TGT TT-3′), and GapA_forward and GapA_reverse (Blumer *et al*., 2005) were used to amplify EHEC-*hlyA*, *espP* and *gapA* (encoding d-glyceraldehyde-3-phosphate dehydrogenase A) used as a reference respectively. The one-step RT-PCR included a reverse transcription step at 42°C for 10 min, and a polymerase activation and preliminary denaturation step at 95°C for 10 min, followed by 35 cycles of denaturation at 95°C for 15 s, annealing at 57°C for 15 s and extension at 72°C for 20 s. A melting curve analysis to confirm the specificity of the amplification products was constructed with continuous fluorescence reading from 55°C to 95°C. Data were analysed using the Bio-Rad iQ5 standard edition optical system software V2.0. EHEC-*hlyA* and *espP* mRNA levels were normalized to *gapA* mRNA and compared with the 2 h time point. Quantitative RT-PCRs were performed three times with two independent RNA preparations.

### Cell lysis assay

Cytolytic activity of clones TA142, TA143, TA144 and TA145 was determined using cultured HBMECs (Stins *et al*., 1997), which are sensitive to EHEC-Hly-mediated lysis (Aldick *et al*., 2007). Cell lysis was determined by measuring release of LDH from HBMECs exposed to bacterial cultures or supernatants as described previously (Aldick *et al*., 2007) with slight modifications. Briefly, HBMECs were seeded (1 × 10^4^ cells per well) into 96-well plates in Endothelial medium (PAA, Pasching, Austria) and grown until confluence. To investigate LDH release caused by bacteria, 100 µl of twofold dilutions (3 × 10^8^ to 7.5 × 10^7^ cfu) of overnight bacterial cultures grown in LB broth supplemented with 5 mM CaCl_2_, ampicillin (100 µg ml^−1^) and kanamycin (30 µg ml^−1^) were added to the cells (A) or to empty wells (D) and incubated (37°C, 5% CO_2_) for 4 h. Bacterial cultures were then removed and cells were incubated for additional 18 h (37°C, 5% CO_2_) in cell culture medium supplemented with 5 mM CaCl_2_ and gentamicin (100 µg ml^−1^). Untreated cells (C), cells exposed to 0.9% Triton X-100 (added to the cells 45 min before termination of the incubation) (B) and wells with 100 µl of medium without cells (E) served as controls. To test lytic activity of supernatants, HBMEC monolayers were incubated for 18 h (37°C, 5% CO_2_) with 100 µl of sterile-filtered supernatants of the above cultures diluted 1:2 in cell culture medium using the same controls. After incubation, the plates were centrifuged (250 *g*, 5 min), 50 µl of the samples were transferred into a new microtiter plate and the LDH activity was determined using the CytoTox 96 kit (Promega, Mannheim, Germany) according to the manufacturer's instructions. The absorbance was measured at 490 nm (Dynex microplate reader) and LDH release was calculated as follows: [(A − C) − (E − D)]/(B − C) × 100.

### Statistical analysis

The statistical analysis of results was performed using Student's *t*-test; *P* ≤ 0.05 was considered significant.

## Supporting information

**Fig. S1.** Cleavage and inactivation ofrecombinant EHEC-Hly from EHEC O26 via EspPα from EHECO157.

A and B. Immunoblot analysis using anti-EHEC-Hly antibody of (A)recombinant isolated EHEC-Hly after incubation with buffer (control) or with recombinant purified EspPα, and (B) TCA-precipitated supernatants of clones TA142 and TA143 coexpressing recombinant EHEC-Hly and either EspPα (TA143) or the non-proteolytic EspPα mutant S263A (TA142). The arrows indicate the 107 kDa band of intact EHEC-Hly (white arrow) and the Mr ∼84 kDa and the ∼82 kDa EHEC-Hly cleavage products (black arrows). The hash sign (#) indicates a background signal observed regularly in control EHEC-Hly preparations.

C. Haemolytic activity of sterile culture supernatants of clones TA142 and TA143 after 11 h and 13 h of growth calculated as percentage of haemolysis. Data are presented as means ± standard deviations of three independent assays. Statistically significant differences between haemolytic activity of TA142 and TA143 (*P* < 0.01, Student's *t*-test) are indicated by asterisks.

**Fig. S2.** Chronology of EHEC-*hlyA* and *espP* expression in EHEC strains during growth in LB broth. EHEC strains producing EHEC-Hly together with EspPα (O157 : H7 strain EDL933 and O26 : H11 strain 5236/96) or EspPβ (O6 :HNT strain 3503/98 and O163 : H19 strain 36/03) were grown in LB broth (37°C, 180 r.p.m.) for 2–24 h as indicated. Bacteria were harvested by centrifugation, RNA was isolated and transcription levels of EHEC-*hlyA* and *espP* were determined using RT-PCR and normalized to *gapA*. Upregulation of each gene expression relative to 2 h time point was determined using Student's *t*-test with **P* < 0.05 and ***P* < 0.001.
