# Supplementary figures and images for: Enterohaemorrhagic Escherichia coli haemolysin is cleaved and inactivated by serine protease EspPα
Source: Environ Microbiol. 2011 May;13(5):1327–41. doi: 10.1111/j.1462-2920.2011.02431.x (PMC3472028; doi:10.1111/j.1462-2920.2011.02431.x)

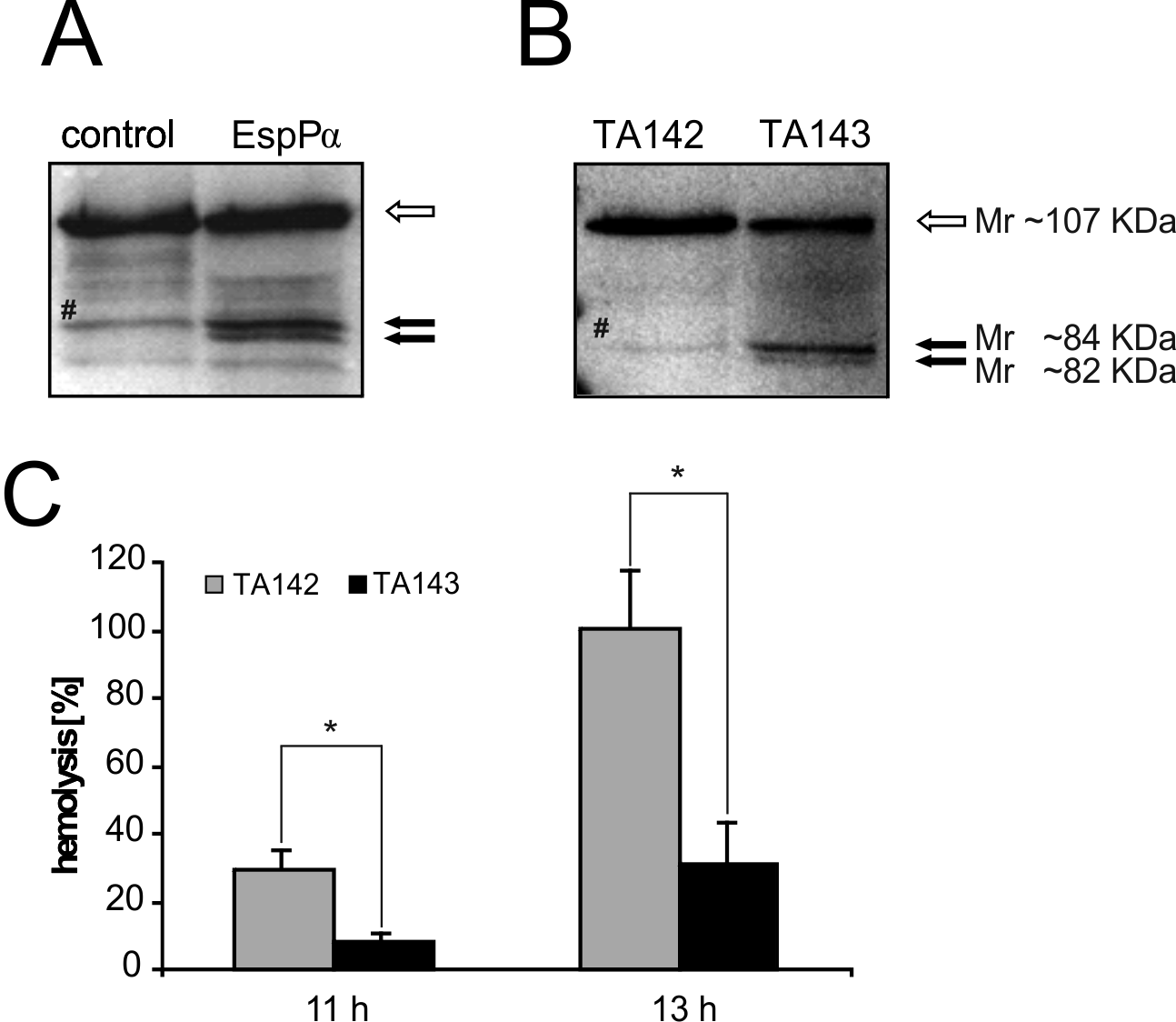

Supplement: Supplementary file 1 [file emi0013-1327-SD1.tif]

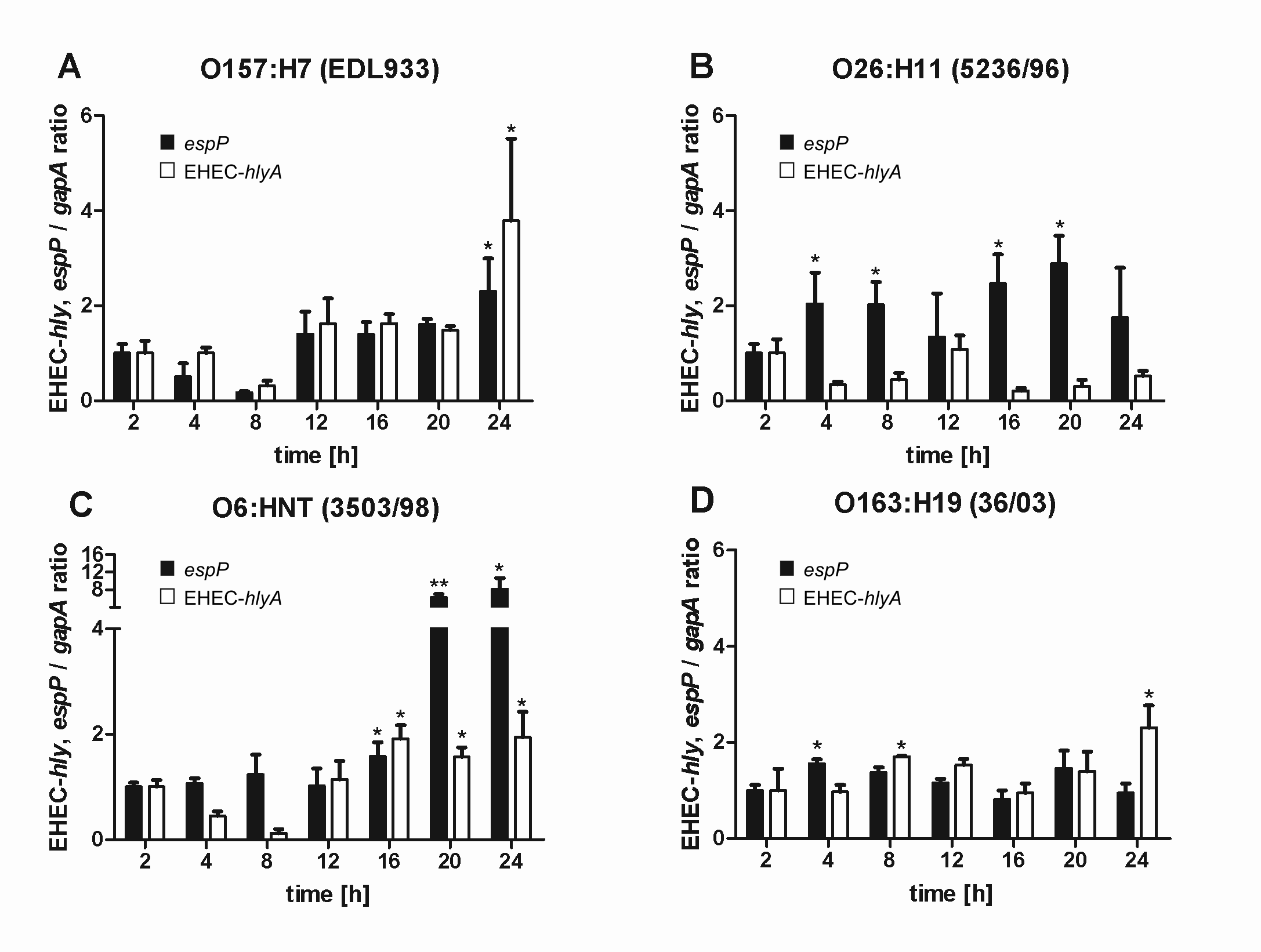

Supplement: Supplementary file 2 [file emi0013-1327-SD2.tif]
